# Missed opportunities for tobacco use screening and brief cessation advice in South African primary health care: a cross-sectional study

**DOI:** 10.1186/1471-2296-11-94

**Published:** 2010-11-29

**Authors:** Olufemi B Omole, Kabilabe NW Ngobale, Olalekan A Ayo-Yusuf

**Affiliations:** 1 Department of Family Medicine, University of the Witwatersrand, Johannesburg, South Africa; 2 Department of Community Dentistry, University of Pretoria, Pretoria, South Africa

## Abstract

**Background:**

Primary health care (PHC) settings offer opportunities for tobacco use screening and brief cessation advice, but data on such activities in South Africa are limited. The aim of this study was to determine the extent to which participants were screened for and advised against tobacco use during consultations.

**Methods:**

This cross-sectional study involved 500 participants, 18 years and older, attended by doctors or PHC nurses. Using an exit-interview questionnaire, information was obtained on participants' tobacco use status, reason(s) for seeking medical care, whether participants had been screened for and advised about their tobacco use and patients' level of comfort about being asked about and advised to quit tobacco use. Main outcome measures included patients' self-reports on having been screened and advised about tobacco use during their current clinic visit and/or any other visit within the last year. Data analysis included the use of chi-square statistics, t-tests and multiple logistic regression analysis.

**Results:**

Of the 500 participants, 14.9% were current smokers and 12.1% were smokeless tobacco users. Only 12.9% of the participants were screened for tobacco use during their current visit, indicating the vast majority were not screened. Among the 134 tobacco users, 11.9% reported being advised against tobacco use during the current visit and 35.1% during any other visit within the last year. Of the participants not screened, 88% indicated they would be 'very comfortable' with being screened. A pregnancy-related clinic visit was the single most significant predictor for being screened during the current clinic visit (OR = 4.59; 95%CI = 2.13-9.88).

**Conclusion:**

Opportunities for tobacco use screening and brief cessation advice were largely missed by clinicians. Incorporating tobacco use status into the clinical vital signs as is done for pregnant patients during antenatal care visits in South Africa has the potential to improve tobacco use screening rates and subsequent cessation.

## Background

Tobacco is the single most significant cause of preventable morbidity and mortality globally[1]. The last South African Demography and Health Survey (SADHS) of 2003 reported a cigarette smoking prevalence of 35% among men and 10% among women. In the same report, 3% of men had ever used smokeless tobacco (SLT), compared to 12% of women. Although fewer Black South Africans smoke cigarettes compared to other racial groups, the majority of SLT users were Black women[2].

The health implications of tobacco use are well documented and include deaths attributable to direct smoking, passive smoking and the use of SLT[3]. Smoking currently accounts for 8% to 9% of all deaths in South Africa; and tobacco use is ranked third, after unsafe sex and high blood pressure, as the most important of 17 evaluated risk factors for mortality in South Africa[4]. Irrespective of the form in which users use tobacco, complete cessation reduces the risks associated with tobacco use[5-8].

Primary health care (PHC) is the most common setting for the provision of tobacco cessation advice[9]. Clinicians at this level of health care are well placed to use every patient contact as an opportunity for screening patients for and advising them against tobacco use. A study in the USA found that physicians knew the smoking status of their patients in only 66% of patient visits; and generalists felt more confident and more frequently offered cessation advice than specialists (22% vs 10%)[10]. In another study in the USA, the counseling behavior of physicians was associated with clinicians' perception of clinical relevance[11]. In the South African PHC context, however, the available literature (which is very limited) suggests that doctors do not intervene in their patients' tobacco use habits[12].

Brief cessation advice improves cessation rates [13] and has been recommended as part of the clinical practice guideline for the management of tobacco dependence[14]. The World Health Organization's (WHO) Framework Convention for Tobacco Control (FCTC) - of which South Africa is a signatory, expects signatories to provide tobacco dependence treatment/cessation services[15]. Although patients consider their family doctor a key influence and a source of advice on smoking,[16] many doctors focus on the presenting clinical problem(s) and are reluctant to use their influence to encourage health-promoting behaviors in the absence of disease, including intervening in a patient's tobacco use[12]. This behavior has serious implications for South Africa, in that its overstretched health system (mainly due to the HIV/AIDS and TB pandemics) can hardly afford the additional increased burden of tobacco-attributable diseases.

Tobacco use is highly prevalent in South Africa. It thus necessitates intensive and sustained interventions, which include ongoing PHC screening and counseling. The clinical consultation in PHC provides opportunities for these activities. However, PHC clinicians' tobacco use screening behavior is hardly documented in South Africa and the only available literature suggests that doctors in South Africa are not intervening in their patients' tobacco use habits[12]. This article reports on the extent to which tobacco use screening is done and the proportion of tobacco users provided with cessation advice in a South African PHC setting, highlighting the implications for tobacco control in the country.

## Methods

### Design and research setting

This cross-sectional study was conducted among patients 18 years and older attending a large Community Health Centre in South Africa during September 2008. This health facility provides a full range of curative services (including obstetric services), health promotion and rehabilitation services to a target population of approximately 75 000 and manages about 5 500 adult patients per month. The health care services are provided by six PHC nurse clinicians and three PHC doctors. In this facility, nurse clinicians dispense medication in their consulting rooms, while doctors' prescriptions are dispensed by pharmacists in the Centre's pharmacy.

### Recruitment of participants' and consent

A sample size of 360 was calculated to be adequate, based on a margin of error of 5%, a confidence level of 95% and a response distribution of 50%[17]. However, 500 participants were recruited to compensate for possible incomplete data.

Consecutive patients were invited to participate in the study after they had exited from the consulting rooms where they were attended to. Patients attended by nurses, and doctors' patients whose visits did not result in prescriptions were processed in a room next to the one where patients return their files, to minimize interruptions in patient flow. Patients attended by doctors who had written prescriptions were processed in the pharmacy waiting room to minimize interruptions and delays in patient flow. In both instances, participants were given a participant information leaflet to read. Trained research assistants explained the leaflets to patients who were not literate. Participants who agreed to participate in the study then completed a consent form. Thereafter, the questionnaire was administered. Recruitment continued over a period of one week, when the required 500 participants were recruited. Critically ill patients, those younger than 18 years old and those who did not give consent were excluded. In order to minimize contamination from staff, only the facility manager and the research team were aware of the nature of the study. All the attending clinicians were blinded to the nature and aims of the study. Recruitment and data collection were also done after the patients had exited from the consulting rooms and at locations away from the consulting rooms. In addition, participants were advised not to discuss the study with any staff members.

### Data collection

The five "A"s (Ask, Assess, Advise, Assist, Arrange) summarize the roles of health care practitioners in managing smoking[18]. A semi-structured questionnaire was adapted from the Patient Exit Interview questionnaire, which has been validated as a good measure of providers' behaviour on smoking cessation intervention[19]. In order to focus on the objectives of the study, only two of the five "A"s (Ask and Advise) were covered by the current questionnaire (Additional file 1). The questionnaire collected information on participants' demographics, their use of tobacco products, their intention to quit tobacco use, participants' reports on having been screened for tobacco use (or not) and clinicians' offer of cessation advice (if any). The main presenting health problem was extracted from the participants' medical records. Primary outcome measures were participants' self-reports of being screened and advised to quit tobacco use by the attending clinician, during the current clinic visit and during any other visit in the last year.

### Data analysis

Descriptive statistics of participants' characteristics were calculated, with categorical data presented as percentages and continuous data presented as means with standard deviations. Socio-demographic factors associated with tobacco use status and the reporting of screening were determined using t-tests (for continuous data), the chi-square test (for categorical data) and Fisher's exact test, where the expected cell count was less than 5. In bi-variate analysis, the associations between the presenting health problems and being screened during the current clinic visit or during a prior visit within the last year were determined. Significant correlates of tobacco use and tobacco screening in all bi-variate analyses were then entered into two separate multiple logistic regression models, in order to determine factors which are independently associated with being screened for tobacco use during the current visit or prior clinic visits within the last year. Group differences were considered statistically significant when p values were lower than 0.05.

### Ethics and permission

Ethics clearance was obtained from the Human Research Ethics Committee of the University of the Witwatersrand [Protocol number M080514], and permission to conduct the study was granted by the Sedibeng District Health Services.

## Results

Five hundred (500) participants completed the questionnaire, of whom 89% (n = 444) were Black and 11% (n = 55) were from other population groups, mainly White. Of the participants, 26.8% (n = 134) were current tobacco users and 6.5% were ex-users. Further analysis showed that 14.8% (n = 74) were current smokers (37.7% of men and 7.5% of women) and that 12.0% (n = 60) were SLT users (1.6% of men and 15.4% of women). The mean number of cigarettes smoked daily was 7.3, while SLT was used about 2.8 times per day. Other characteristics of the participants are shown in Table 1.

**Table 1:** Study participants' characteristics (NB: All n did not add up to 500 because of missing data)

Characteristics	%(n)	Mean (SD)
**Age (years)**		47.9 ±17.0
		
**Sex**		
*Female*	75.5 (376)	
*Male*	24.5 (122)	
		
**Race**		
*Blacks*	89.0 (444)	
*Other (non-Blacks)*	11 (55)	
		
**Marital status**		
*Single/never married*	35.6 (176)	
*Divorced/separated/widowed*	19.0 (94)	
*Married*	45.3 (224)	
		
**Educational achievement**		
*Less than Grade 12*	75.2 (375)	
*Grade 12 and above*	24.8 (124)	
		
**Current cigarette smokers**	14.8 (74)	
		
**Current snuff users**	12.0 (60)	
		
**Number of cigarettes per day**		7.3 ±5.9
		
**Smokeless tobacco use per day**		2.8 ±1.7
		
**Presenting health problem**		
*Cardiovascular disease*	50.2 (250)	
*HIV-related care*	12.2 (61)	
*Pregnancy-related care*	18.5 (92)	
*Other*	19.1 (95)	
		
**Attending clinician**		
*Medical practitioner*	57.7 (287)	
*Nurse clinician*	42.3 (210)	

Only 12.9% (n = 63) of the participants reported having been screened for tobacco use during the current visit and 10.6% (n = 53) reported that they had been screened during prior visits within the last year. Of those who reported that they had been screened during the current visit, 10.4% (n = 14) were current tobacco users. The proportions of tobacco users who reported having been screened during the current or prior clinic visits were not significantly different from the proportions of non-users who reported having been screened at similar clinic visits (see Table 2).

**Table 2:** Socio-demographic correlates of tobacco use and reporting receiving tobacco screening

Socio-demographic variable	% (n) Smoking	% (n) Snuff	%(n) screened during current visit	%(n) screened during a prior visit
**Current visit screening**				
No	15.0 (64)	12.9 (55)	-	
Yes	15.9 (10)	6.3 (4)	-	
p =	0.86	0.14		
**Prior visit screening**				
No	14.6 (65)	12.6 (56)	10.6 (46)	-
Yes	17.0 (9)	7.5 (4)	32.1 (17)	-
p =	0.65	0.38	< 0.001	-
**Sex**				
Female	7.4 (28)	15.4 (58)	14.1 (52)	11.2 (42)
Male	37.7 (46)	1.6 (2)	9.1 (11)	9.1 (11)
p =	< 0.001	< 0.001	0.15	0.52
**Race**				
Black	11.9 (53)	13.5 (60)	13.3 (58)	9.5 (42)
Other	38.2 (21)	0	9.3 (5)	20.0 (11)
p =	0.49	0.01	0.40	0.02
**Educational level**				
Less than Grade 12	15.5 (58)	14.4 (54)	9.6 (35)	8.3 (31)
Grade 12 and higher education	12.9 (16)	4.8 (6)	22.6 (28)	17.7 (22)
p =	0.49	0.01	< 0.001	< 0.01
**Marital status**				
Single	11.9 (21)	10.8 (19)	18.4 (32)	15.3 (27)
Separated/divorced/widowed	20.2 (19)	14.9 (14)	7.8 (7)	4.3 (4)
Married	14.7 (33)	11.2 (25)	10.9 (24)	9.9 (22)
p =	0.19	0.57	0.02	0.02
**Current smoker**				
No	-		12.7 (53)	10.4 (44)
Yes	-		13.5 (10)	12.2 (9)
p =			0.86	0.65
**Snuff user**				
No		-	13.7 (59)	11.2 (49)
Yes		-	6.8 (4)	6.7 (4)
p =			0.21	0.38
**Attending clinician**				
Medical practitioner	20.2 (58)	11.0 (23)	5.7 (16)	8.0 (23)
Nurse clinician	7.6 (16)	12.5 (36)	22.8 (47)	14.3 (30)
p =	0.001	0.59	< 0.001	0.03
**Presenting health problem**				
HIV-related problem	14.8 (9)	14.8 (9)	10.0 (6)	9.8 (6)
Pregnancy-related problem	3.3 (3)	3.3 (3)	40.7 (37)	22.8 (21)
Cardiovascular disease	14.8 (37)	14.4 (36)	3.7 (9)	6.8 (17)
Other	26.3 (25)	12.6 (12)	11.6 (11)	8.4 (8)
p =	< 0.001	0.04	< 0.001	< 0.001

Of the participants who had not been screened, 88% (n = 396) indicated they would be 'very comfortable' with being screened for tobacco use. Although 78.3% (n = 94) of tobacco users said they intended to quit tobacco use in the next six months, the proportion of tobacco users at this stage of behavioral change was not significantly associated with reporting being screened or not screened during the current visit (66.7% vs. 79.4%; p = 0.29), nor with reporting being screened or not screened during prior visits in the last year (55.6% vs. 80.2%; p = 0.20). Reporting being screened during the current visit was also associated with reporting being screened within the last year (p < 0.001). Other socio-demographic correlates of tobacco use and screening are shown in Table 2.

Of the 134 current tobacco users, 11.94% (n = 16) reported being advised about the cessation of tobacco use during the current clinic visit, while 35.1% (n = 47) reported being advised about it during prior visits within the last year. Of the 47 tobacco users who reported that they had been advised during prior clinic visits, 13 (27.7%) reported having been advised again during the current clinic visit. Among tobacco users, 81.5% (n = 101) reported that it was important to be advised against tobacco use, while 85.9% (n = 110) reported that they thought providing cessation advice could assist them in quitting tobacco use.

In the final logistic regression models (Table 3), being screened during a prior clinic visit in the last year remained independently associated with tobacco use screening during the current visit (OR = 2.65; 95%CI = 1.26-5.54). Compared to any other presenting health problems, a pregnancy-related visit was the single most significant factor associated with being screened for tobacco use during the most recent clinic visit (OR = 4.59; 95%CI = 2.13-9.88).

**Table 3:** Final logistic regression model of factors associated with receiving tobacco use screening

Explanatory variable	Screened during current visit [OR (95% CI)]	Screened during prior visit [OR (95% CI)]
**Age (continuous)**	-	0.96 (0.95 - 0.98)
**Race**		
Non-Black	-	1
Black		0.24 (0.11 - 0.54)
**Presenting health problem**		
Other	1	
Cardiovascular disease	0.30 (0.12 - 0.75)	-
HIV-related problem	0.85 (0.29 - 2.45)	
Pregnancy-related visit	4.59 (2.13 - 9.88)	
**Past screening experience**		
Never/rarely	1	
Screened at prior visits	2.65 (1.26 - 5.54)	-
	***n = 488, Nagelkerke R^2 ^= 0.27*** ***Model fit (Hosmer & Lemeshow X^2^) P = 0.91***	***n = 497, R^2 ^= 0.11*** ***Model fit (Hosmer & Lemeshow X^2 ^test) = 0.97***

## Discussion

This study is one of very few studies documenting patients' report of clinicians' behavior regarding tobacco cessation activities in a PHC setting in South Africa. The results show that opportunities provided by the clinical encounter for screening and providing tobacco use cessation advice were largely missed by clinicians. The results confirm the findings of a qualitative study of health care practitioners elsewhere in South Africa, which found that few clinicians addressed their patients' tobacco use habits[12]. The lack of doctors' intervention in patients' tobacco habits in South Africa is contrary to what pertains in other countries such as Australia, the USA and China, where physicians are reported to be more engaged in activities targeting patients' tobacco use[20-22]. The poor engagement of clinicians with tobacco intervention activities may reflect clinicians' failure to recognize the potential for such interventions within the clinical consultation in reducing rates of tobacco use, in addition to those already achieved through policy interventions such as tax increases and advertisement bans in South Africa. Intensifying clinicians' engagement in tobacco cessation activities in South Africa is crucial for a successful tobacco control program, especially since the report of the recent SADHS found that the prevalence of tobacco use among men has been only slightly reduced and has remained virtually stagnant among women and young people since the last SADHS in 1999[2].

Brief advice during clinical encounters (typically no longer than 5 minutes) has been shown to be more effective than "no advice" in increasing quitting rates among tobacco users [12]; and the role(s) of health professionals in helping their patients to quit is well recognized[16]. However, a large proportion of participants in this study were not advised against tobacco use, possibly due to barriers which have previously been documented [15,16], namely time constraints, a lack of counseling skills, a lack of guidelines and protocols, the absence of economic re-imbursement, the belief that brief advice is not effective, the perception that it is not a clinician's responsibility to help clients to quit, the belief that patients are not motivated to quit and reluctance to jeopardize the doctor-patient relationship by giving advice perceived to be unwelcome[16,23-25]. Whether these barriers apply in the South African context is not yet known and further studies are needed to determine this. Nevertheless, training health care practitioners in tobacco cessation counseling is crucial and has been shown to improve both counseling rates and the quality of support given to patients who want to quit cigarette smoking[26,27].

Regular tobacco use screening and brief advice should result in more participants' contemplating quitting, but the findings of this study do not support this assumption. Screening (during the current visit or in the last year) was not found to be significantly associated with a state of contemplating quitting (p = 0.21 and p = 0.09 for the current and the last year respectively). A possible explanation for this observation could be that when clinicians screen patients they do not follow up with cessation advice, a key motivation for tobacco users to contemplate quitting tobacco use.

The vast majority (88%) of the participants who had not been screened stated that they would be "very comfortable" with tobacco use screening, while 78.3% of tobacco users intended to quit within the next six months. These findings are similar to findings from previous national surveys in South Africa [28] and they highlight the scale of missed opportunities in intervening in patients' tobacco use.

The majority of tobacco users (81.5%) knew the importance of being advised and reported that being advised could help them quit tobacco use. Since smokers have been documented to respond favorably to their physicians' advice, [29] not screening patients for and advising patients against tobacco use constitute serious omissions, with both clinical and public health implications. Screening and providing brief cessation advice improve the probability of patients' quitting tobacco use and has the potential to decrease the several thousand deaths from tobacco use, allowing for a rechanneling of resources expended on the treatment of tobacco-related diseases to other more pressing health needs, such as HIV and AIDS treatment. These gains of the anti-tobacco campaign at the public health level are being lost due to these missed opportunities in the clinical setting.

Given the well-documented adverse effects of tobacco on the fœtus and the concerning report that up to 20% and 10% of South African women smoke or use SLT respectively during pregnancy,[15,30], it is a welcome finding that participants attending the Centre for pregnancy-related conditions were more likely to have been screened for tobacco use, compared to those presenting with any other condition. Although pregnant patients were attended to almost exclusively by midwives in the current research setting, it would appear that the usual practice of documenting (checking off) patients' tobacco use status on the antenatal card during pregnancy-related visits is responsible for the above finding, rather than the grade of the attending clinician. In fact, the grade of the attending clinician was not significantly associated with reporting being screened when potential confounders were controlled for. Including tobacco use status as a vital sign (as done on the antenatal card) has indeed been shown to improve both tobacco use screening and clinicians' offer of cessation advice[31]. Therefore, setting up systems such as these that prompt clinicians to document patients' tobacco use status may be an important strategy that could be considered in the South African PHC settings for the promotion of tobacco screening and intervention[32].

Illnesses offer "teachable" moments, during which patients heed advice more readily and the knowledge of serious diagnoses such as cardiovascular diseases results in higher quit rates for tobacco use[33,34]. Despite the fact that more than half (50.2%) of the participants in the current study consulted their clinicians for cardiovascular-related problems, this diagnosis was not associated with reporting being screened for tobacco use. This is a cause for serious concern in South Africa, as the country is currently undergoing a disease transition in which there is an increase in the burden of cardiovascular diseases. In addressing this concern, it should be recognized that the monthly visits of chronic patients to their PHC clinics (for check-ups, collection or renewal of medications) provide opportunities for screening for, advising and reinforcing previous advice on tobacco cessation. Unfortunately, these opportunities were largely missed by clinicians in this study. Stopping tobacco use is a difficult endeavor and often requires many counseling attempts. Reinforcing cessation advice as a way of intensifying the delivery of counseling is a strategy that has been shown to improve the cessation success rate [14] and should be employed to enhance the odds of patients' successfully quitting. Regrettably, patient contacts were not used to reinforce previous advice, as only just over a quarter of the participants who had been advised to quit in the last year were re-advised to do so during the current clinic visit.

The cessation of all forms of tobacco use improves health outcomes. Thus interventions which enable tobacco users to achieve life-long abstinence must be regarded as clinical and public health priorities[35]. While screening and advising do not guarantee successful quitting, they assist clinicians in identifying tobacco users and provide platforms for further cessation interventions, such as providing more intense advice and referring to specialized services or national quit-lines.

The convenience sample used in this study may not be representative of the entire clinic population, because of possible selection bias. Since this study did not use a nationally representative sample, caution must be exercised in generalizing the findings of this study to all patient visits in South Africa. However, the rates of tobacco use among participants in the current study approximate recent national estimates[2]. In the current study, the smoking prevalence among men was slightly higher at 37.7%, compared with a national prevalence of 35%. The smoking prevalence among women was however, slightly lower at 7.5%, compared to a national estimate of 10%. Similarly, 1.6% of men (compared to a national prevalence of 3%) and 15.4% of women (compared to a national prevalence of 12%) used SLT.

The current study focused only on two of the 5 A's of tobacco cessation interventions (Ask and Advise), because of the exploratory nature of the study, and the recognition that many clinicians in South Africa do not yet provide comprehensive clinical interventions for tobacco users. Barriers to cessation counseling and the extent to which these barriers influence clinicians' behaviors were not investigated. These will be the focus of a qualitative study currently being conducted by the authors. Given that people tend to report favorable behavior when they are interviewed about a socially undesirable behavior such as tobacco use, reliance on participants' self-reports can potentially lead to information bias and a high rate of misclassification. Considering the objectives of the current study, we do not think that changing the study design will produce significantly different results from the ones we have reported in this article. Notwithstanding these limitations, this study (one of the very few documenting PHC clinicians' tobacco screening and advising behaviors) provides useful information to develop tobacco cessation programs and to study tobacco cessation in South Africa further.

## Conclusion

Despite participants' claim that they would be comfortable with tobacco use screening and cessation advice, only a small proportion of the participants were screened and advised against tobacco use. Regular clinic visits were not used to reinforce previous tobacco cessation advice among tobacco users. The magnitude of missed opportunities documented in this study threatens tobacco control efforts in South Africa and undermines the important role that PHC practitioners can play in further reducing the prevalence of tobacco use in South Africa.

Pregnancy-related visits increased the likelihood of being screened for tobacco use, probably because tobacco use status is included in the list of vital signs on the antenatal card in South Africa. Extending this strategy to all PHC patient-visits has the potential to compel clinicians to screen all patients for tobacco use and may consequently improve screening and intervention rates.

Further studies which focus on understanding why PHC clinicians in South Africa do not intervene in their patients' tobacco use are needed in order to implement effective tobacco cessation programs.

## Competing interests

The authors declare that they have no competing interests.

## Authors' contributions

OB was involved in the conception, design, data collection, and interpretation of the results, as well as the drafting of the manuscript, and gave approval for this version to be published. KNW was involved in the conception, design, data collection, and interpretation of results, and gave approval for the final version to be published. OA was involved in the conception, design, data analysis, and interpretation of results, as well as in revising the manuscript, and gave final approval for the final version to be published.

## Authors' information

OB is Head of Clinical Unit (Family Medicine) at Sedibeng District Health Services, Gauteng Department of Health, South Africa, in joint appointment with the Department of Family Medicine, University of the Witwatersrand, South Africa

KNW is Senior Specialist (Family Medicine) at Sedibeng District Health Services, Gauteng Department of Health, South Africa, in joint appointment with the Department of Family Medicine, University of the Witwatersrand, South Africa

OA is an Associate Professor in the Department of Community Dentistry, University of Pretoria, South Africa, in joint appointment with the Gauteng Department of Health, South Africa.

## Pre-publication history

The pre-publication history for this paper can be accessed here:

http://www.biomedcentral.com/1471-2296/11/94/prepub

## Supplementary Material

Additional file 1 **Patient exit questionnaire**. The questionnaire used in this current study. Click here for file
