# Supplementary material for: Missed opportunities for tobacco use screening and brief cessation advice in South African primary health care: a cross-sectional study
Source: BMC Fam Pract. 2010 Nov 29;11:94. doi: 10.1186/1471-2296-11-94 (PMC3009621; doi:10.1186/1471-2296-11-94)
Supplement: Additional file 1 — Patient exit questionnaire. The questionnaire used in this current study. [file 1471-2296-11-94-S1.DOCX]

**TOBACCO RESEARCH QUESTIONNAIRE**

Patient code: ................................

Patient seen by a doctor [ ] Patient seen by a PHC Nurse [ ]

**A. DEMOGRAPHICS**

1. Age: ………years

2. Sex: Male [ ] Female [ ]

3. Marital Status:

- Divorce [ ]

- Single [ ]
- Married [ ]
- Widowed [ ]

4. Occupation:

- Artisan [ ]

- Pensioner [ ]
- Professional [ ]
- Student [ ]
- Unemployed [ ]

5. Level of education:

- Below matric [ ]

- Matric [ ]
- No schooling [ ]
- Post matric diploma [ ]
- University degree [ ]

6. Race:

- African [ ]

- White [ ]
- Coloured [ ]
- Indian [ ]
- Others [ ]

7. Diagnosis OR Clinical problem(s):…………………………………………………………………………………………………………………………………………………………………………………

**B. PARTICPANTS’ USE OF TOBACCO**

8. Do you currently use any form of tobacco: Yes [ ], Not currently [ ], Never [ ]

**(IF ANSWER OTHER THAN “YES” TO QUESTION 8, SKIP TO QUESTION 12)**

9. If Yes to question number 8,

What type(s) of Tobacco do you currently use? **Select all that applies**

- Chewing tobacco leaves [ ]

- Cigarette smoking [ ]
- Snuff [ ]
- Others [ ] Specify:…………………………………………………

10. How long have you been using this tobacco product you mostly use currently?………………………(Years)

1. What amount of this tobacco do you use in one day?

--------- number of sticks of cigarette per day

---------- number of times snuff is used per day

--------- number of times tobacco leaves are chewed per day

Others: please specify----------------------------------------------- per day

**C. ENQUIRY ABOUT THE USE OF TOBACCO**

12. How frequently do clinicians ask you about your tobacco habit?

- During all visits [ ]
- Most of the time [ ]
- Rarely [ ]

- Never [ ]

1. During today’s consultation, were you asked about your use of tobacco?

Yes [ ] No [ ]

If Yes, how did you feel about your being asked about your use of tobacco?

- Not comfortable [ ]

- Somewhat comfortable [ ]

- Very comfortable [ ]

14. If your response to question (13) was **No**,

How would you have felt, if the clinician asked you about your use of tobacco:

- Not all comfortable [ ]
- Somewhat comfortable [ ]

- Very comfortable [ ]

**D. TOBACCO CESSATION ADVICE *(SKIP IF NOT CURRENTLY USING ANY TOBACCO PRODUCT)***

15. Have you been advised on your tobacco habit in the past one year?

Yes [ ] No [ ]

16. If you were asked about your tobacco habit during today’s consultation, were you advised on stopping the use of tobacco?

Yes [ ] / No [ ]

- If yes, what were you told?……………………………………………………….

…………………………………………………………………………………………………………………………………………………………………………………

- If you were not advised, do you think it was important to have been advised: Yes [ ] / No [ ]

Give reason(s) for your response………………………………………………….

……………………………………………………………………………………….……………………………………………………………………………………………

1. Do you think advising you could assist you in quitting the use of tobacco?

Yes [ ] No [ ]

- If yes, why do you think so? ---------------------------------------------------------------------------------------------------------------------------------------------------------------------------------------------------------------------------------------------------------------------------------------------………………..
- If No, why do you think so?------------------------------------------------------------------------------------------------------------------------------------------------------------------------------------------------------------------------------------------------------------------------------------------------------------------

18. Were you thinking of quitting the use of tobacco in the next six months? Yes [ ] No [ ]

***THANK YOU FOR YOUR TIME***
